# The athlete’s heart: insights from echocardiography

**DOI:** 10.1186/s44156-023-00027-8

**Published:** 2023-10-18

**Authors:** Harry Flanagan, Robert Cooper, Keith P. George, Daniel X. Augustine, Aneil Malhotra, Maria F. Paton, Shaun Robinson, David Oxborough

**Affiliations:** 1 Research Institute for Sport and Exercise Sciences, Liverpool John Moores University, Tom Reilly Building, Byrom Street, Liverpool, L3 3AF UK; 2 Royal United Hospitals Bath NHS Foundation Trust, Bath, UK; 3 Institute of Sport, Manchester Metropolitan University and University of Manchester, Manchester, UK; 4 Leeds Institute of Cardiovascular and Metabolic Medicine, University of Leeds, Leeds, UK; 5 Imperial College Healthcare NHS Trust, London, UK; 6 Department for Health, University of Bath, Bath, UK

**Keywords:** Athlete’s heart, Echocardiography, Pre-participation screening, Sports cardiology

## Abstract

The manifestations of the athlete’s heart can create diagnostic challenges during an echocardiographic assessment. The classifications of the morphological and functional changes induced by sport participation are often beyond ‘normal limits’ making it imperative to identify any overlap between pathology and normal physiology. The phenotype of the athlete’s heart is not exclusive to one chamber or function. Therefore, in this narrative review, we consider the effects of sporting discipline and training volume on the holistic athlete’s heart, as well as demographic factors including ethnicity, body size, sex, and age.

## Background

Exercise training is associated with an array of morphologic and functional cardiac adaptations and termed the ‘athlete’s heart’ (AH) [1]. These manifestations can include an increase in the left and right cardiac cavity sizes, increased left ventricular (LV) wall thickness and an augmentation in indices of resting and exercise systolic and diastolic function at rest and during exercise, when compared to that of non-athletic individuals. The phenotype of the AH is a whole heart adaptation rather than being exclusive to one chamber or function of the heart. Therefore, when differentiating between the AH and cardiomyopathies it is crucial to evaluate all the cardiac chambers, assessing whether ventricular or atrial dilatation occurs in the context of a global remodeling [2].

**Fig. 1:**
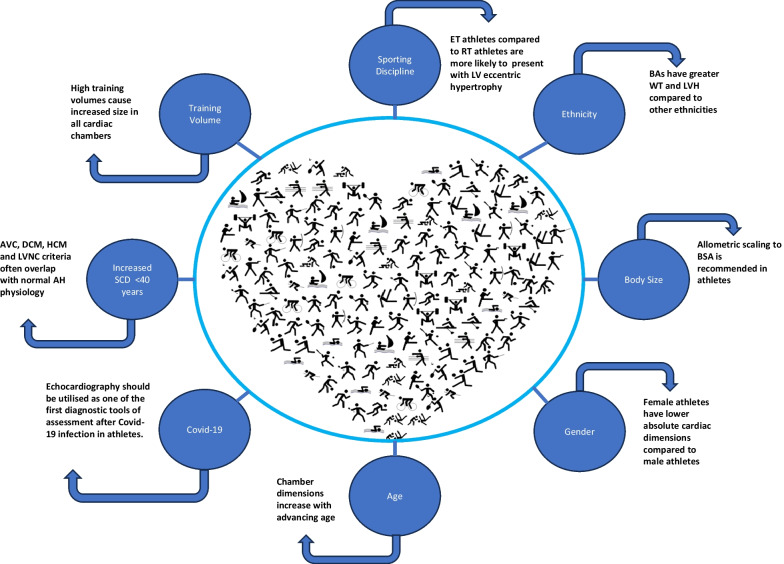
Multifactorial nature of the athlete’s heart

The multifactorial nature of AH morphology (Fig. 1) and functional changes linked with prolonged training exposure (> 4 h per week) [3] can create a diagnostic challenge during echocardiographic assessment. The extent of athletic adaptation often extends beyond ‘normal’ limits of cardiac dimensions and function, making it challenging to differentiate between AH physiology and inherited conditions such as hypertrophic cardiomyopathy (HCM), arrhythmogenic (right ventricular) cardiomyopathy (AVC), dilated cardiomyopathy (DCM) and isolated left ventricular non-compaction (LVNC) [4, 5]. However, in all of the studies that demonstrate physiological hypertrophy there is no evidence of asymmetrical hypertrophy [6]. Consequently, diagnosing a condition that may increase the risk of sudden cardiac death (SCD) in an athlete depends on the precise understanding of the clinical context and the physiological parameters of acceptable change in cardiac morphology [1]. Hence, there is an emphasis placed by scientific organisations and sporting bodies for athletes to undergo routine screening to help identify these conditions. The aim of this review is to advance our knowledge and understanding of the extent of the AH phenotype by assessing thematically the impact of sporting discipline and training volume, ethnicity, body size, sex, and age on the AH.

### Sporting discipline and training volume

#### The left ventricle

Numerous studies have attempted to quantify the extent of left ventricular (LV) adaptation/remodelling and its association with the type of exercise stimulus. The first recognised proposal from this research was the development of the Morangroth hypothesis over 45 years ago. This stated that endurance trained (ET) athletes exhibit eccentric LV hypertrophy whereas resistance trained (RT) athletes exhibit concentric LV hypertrophy [7]. Eccentric hypertrophy is characterised by increased LV mass and end-diastolic volume (LVEDV) and normal wall thickness (WT), while concentric LVH is characterised by increased LVWT and mass but normal LVEDV [7, 8]. Over time, aided by advancements in imaging technology, subsequent studies have confirmed but also disputed the findings by Morganroth.

There is a widespread agreement that ET elicits LV dilation with increased LV mass, LVEDV and LV end-diastolic diameter (LVEDD), whereas neither concentric hypertrophy nor remodelling are now thought to be expressed in RT athletes [9–16]. Utomi et al. have further supported this claim, finding that normal geometry was predominant in both ET and RT athletes with 30% of the ET athletes expressing eccentric hypertrophy [17]. Longitudinal studies have provided disparate findings with one study demonstrating increased LV mass in 11% and 12% of ET and RT athletes respectively and associated concentric geometry in the RT athletes [18]. This concentric hypertrophy in the RT after 3–6 months of training contrasted with a lack of structural changes in subsequent studies in RT athletes over a six-month period [14, 19]. It should be noted however, that with a timeframe of nearly half a century between the first and more contemporary studies, discrepancies within the findings can be accounted for by the differences in training types/volumes but also in the variations in advancing imaging quality and technology [20]. Furthermore, with the ‘modern athlete’ now completing a combination of both endurance and resistance training coupled by the isometric and isotonic components demanded from exercise and competition, the original binary subclassifications of athletes being either purely ET or RT is no longer applicable [1, 20].

A more recent cross-sectional study on 139 elite Rugby Football League (RFL) athletes found that values for LV mass and mean WT were greater in RFL athletes compared to sedentary controls (191 ± 31 g, 132 ± 24 g and 9 ± 1 mm, 8 ± 1 mm respectively) [21]. However, despite the increased LV size, a predominantly normal LV geometry was demonstrated within the RFL athlete group [21]. This was further confirmed in a previous review of the literature which states that only a very small percentage of athletes express a WT above 13 mm [4]. However, it does recognise that some athletes undertaking specific endurance disciplines, such as cycling and rowing, have LV end-diastolic internal dimension (LVIDd) values in excess of 60 mm [4]. Wundersitz et al. investigated this further using a systematic review and meta-analysis comparing cyclists to other disciplines of exercise [16], reporting that endurance cyclists were found to have significantly larger LVEDD than athletes of other categories [16]. However, these findings are in association with a larger heart relative to body size and therefore an increased incidence of diagnostic uncertainty between physiological and pathological adaptation [16]. These findings further support a sport specific response that should be considered.

Recent studies have focussed on examining the effects of varying training volume and intensity on the AH. It has been found that athletes with high levels of training volume showed a more pronounced structural remodelling with significantly higher LV mass and LVEDV than those with a lower training volume per week (99 ± 19 g/m^2^ vs. 90 ± 14 g/m^2^; 160 ± 24 ml vs. 129 ± 32 ml respectively) [22]. Additionally, when comparing high-intensity interval training to moderate intensity continuous training, it has been found that high-intensity interval training induces greater increases in LV mass and that uniquely was the only training method to elicit eccentric hypertrophy [23]. This denotes the high variability of adaptation of the AH, with training intensity and volume having profound effects on structural morphology.

Lower resting LV ejection fraction (EF) in endurance athletes has been demonstrated in previous research (12% found to have an LVEF of < 52%) [24]. This can be accounted for by the increased LVEDV in that a larger LV does not require the same contraction force as a smaller chamber to achieve the same SV [20, 25]. Furthermore, 12% of elite cyclists and athletes over a varying range of training regimes were also identified as having a combination of a lower EF and increased LVEDV [26, 27]. It should be noted that these studies were conducted on cyclists however other studies have also found EF to be lower in a small minority of RFL and American-style football players (48% and 49% respectively) [21, 28]. These finding are likely due to the interaction of divergent effects of increased LVIDd and WT on LV function, in combination with differences in physiological demands [21].

The application of strain imaging in the assessment of the AH has increased significantly in tandem with the evolution of this technology. It has been reported that longitudinal strain (GLS) and basal circumferential strain are lower in RT athletes compared to ET athletes [17]. However, no differences in GLS have been found in ET, RFL players, elite cyclists or rowers when compared to sedentary controls or recreational athletes [14, 17, 21, 26]. This was further supported in a meta-analysis in which no athlete-control differences existed for GLS or global circumferential strain [29].

Athletes demonstrate a superior ability to augment systolic function during exercise [30]. This therefore provides a useful in-exercise assessment tool for those who display decreased contractility at rest and can be applied across sporting disciplines [20]. Additionally, elite ET athletes have also been found to have lower LV twist values than controls with competitive RT athletes presenting greater twist [29]. However, lower twist in elite ET athletes has not been demonstrated in competitive level athletes and thus highlights a plausibility for a dose–response relationship between ET level and adaptations in this specific functional parameter [29].

Diastolic function assessment is multiparametric as Doppler parameters are reliant on both atrial and LV pressures [20]. Unsurprisingly, the findings are equivocal, with many studies observing no differences in peak E velocity between sedentary controls and athletes [15]. At odds to this, the ratios of early to late diastolic filling (E/A—blood flow velocity, e′/a′—myocardial velocity) have been increased in ET athletes [20, 31] potentially due to enhanced early diastolic filling [22, 31]. Conversely, a recent study of elite cyclists found decreased E and e` velocities compared to recreational cyclists [26]. This is a novel finding and could indicate a considerable functional reserve with further research being required to identify whether this is exclusive to elite cyclists or whether it is more widespread in the elite ET population.

Although longitudinal data is scarce, some studies have demonstrated that neither ET nor RT elicit changes in global diastolic function in a period of 6-months training [14, 18] whereas three studies demonstrate increased LV peak untwisting and a decreased time to peak untwisting after 3 months of intense rowing training, high intensity interval training and in elite male and female rowers [12, 23, 32]. These equivocal findings warrant further investigation.

#### The right ventricle

Nearly a quarter of SCDs in athletes have been attributable to AVC [20], a condition that has a worse prognosis when the individual partakes in intense or endurance exercise. It is therefore vital to understand the nature and magnitude of physiological training-induced RV remodelling of the AH. Increased RV cavity, outflow and inflow dimensions along with balanced increases in RV mass and volume are demonstrated in ET athletes compared with RT athletes, whereas pure RT athletes appear to have similar chamber dimensions to sedentary individuals [11, 14, 33–38]. A longitudinal study provides robust evidence for increased RV cavity dimensions after 6 months of ET but not following RT [14]. Additionally, RV outflow (40%) and inflow (57%) dimensions were found to be greater than normal ranges among 102 ET athletes, with 28% of this cohort presenting with larger RV outflow tract dimensions greater than the major structural criteria for AVC [37]. Furthermore, RV enlargement was compatible with a diagnosis of AVC in approximately 40% of Olympic athletes participating in skill, power, mixed and endurance sports [39]. Based on this the variable impact on the RV compared to the LV is an important consideration when evaluating the impact of sport on the AH. Over the duration of a 12-month longitudinal study and in 492 male national athletes respectively, a proportional, progressive increase in RV:LV ratio was found in response to high-dynamic training providing further support for disproportionate loading on the RV [5, 33, 37].

Global resting RV function is maintained and almost always normal when assessed by RV fractional area change, Tricuspid Annular Plane Systolic Excursion (TAPSE), RV myocardial tissue velocities S’ and peak RV free wall strain [5, 11, 35, 37, 39, 40]. There are also data to demonstrate higher values for TAPSE and S’ with similar RV global and free-wall longitudinal strain but regional variance i.e. lower basal strain [34]. Others have found a lower resting RV global strain in athletes compared to controls [34, 41]. Regional variation in RV strain has also been demonstrated albeit with reduced apical strain. This variance was associated with absolute chamber size and contributes to a smaller base to apex strain gradient in both septal and lateral walls of the RV [5]. Regardless of the outcomes, those studies that have identified regional heterogeneity did not demonstrate values as low as those seen in AVC patients [30]. Moreso, abnormal patterns of basal strain, including pre-stretch and time to peak strain have not been identified in the athlete [42]. These normal physiological responses to exercise of the RV can therefore highlight a potential diagnostic role of exercise echocardiography in ambiguous cases wherein physiology and pathology overlap [30].

#### The atria

Patients with cardiomyopathy often present with left atrial (LA) dilation due to a combination of increased LV pressures and atrial myopathy [20], yet bi-atrial dilatation and increased atrial volumes are present in athletes engaging in a mixture of endurance disciplines when compared to sedentary controls [43–52]. It has been demonstrated that mild, moderate, and severe LA enlargement is present in 27%, 11% and 4% of international-level rowers [46]. Additionally, a more recent study denotes that amateur marathon runners have larger atria versus controls when compared with ranges for the general adult population; with 56% of these athletes showing increased LA volume when indexed to body surface area (BSA) [47]. This enlargement is strongly correlated with exercise capacity and therefore could be observed as a further manifestation of the AH phenotype [46]. Furthermore, echocardiographic reference values for highly-trained athletes, but not the general population, must be considered for both elite and amateur athletes in order to avoid misdiagnosis of pathological LA enlargement [47].

Although two studies suggest that there are no differences for any atrial strain parameter between athletes of varying dynamic disciplines and sedentary groups several other studies have suggested that LA reservoir strain and bi-atrial strain rate during atrial contraction are lower in athletes compared to sedentary controls [43, 46, 49, 51, 53]. It should be noted however, that the lower values of longitudinal LA strain seen in the athlete are present in only 4% of the population [46] and therefore is considered to be a relatively rare finding. In relation to these disparities, further research should seek to better characterise the impact of training volume and sporting discipline on atrial function.

#### The aorta

Larger aortic root dimensions tend to be present in some athletes compared with sedentary controls, albeit values usually fall within normal clinical cut offs [54]. It is uncertain whether this represents an aortopathy or a normal physiological response to exercise training [55–58]. A study assessing RFL players, aortic size was found to be within normal reference intervals [59]. It is therefore recommended for clinicians who are evaluating athletes that if there is a marked aortic root dilatation present then this is likely representative of a pathological process and not a physiological adaptation to exercise [58]. Additionally, significant ascending aorta dilatation and aortic regurgitation have been found to be uncommon in RT athletes [60] (Table 1).

**Table 1:** Key consideration points regarding sporting discipline and training volume for preparticipation screening

Key points for preparticipation screening
Endurance athletes often present with physiological eccentric hypertrophy of the LV; however as concentric remodelling in any athlete is rare, further investigation is warranted
Endurance athletes typically present normal or decreased systolic function and normal or superior diastolic function compared with the sedentary population, and hence, any deviation from this expected function warrants further clinical investigation
Athletes engaging in endurance disciplines present bi-atrial dilatation and increased atrial volumes which are strongly correlated with exercise capacity
Endurance and RT athletes tend to present larger aortic root size than the non-athletic population. However, these differences in size are not clinically significant and therefore the presence of a dilated aortic root warrants further investigation

### Ethnicity

#### The left ventricle

##### Black athletes

Our knowledge pertaining to the AH and the LV has been predominantly based on Caucasian athletes (WA) with these data being the driving force used to distinguish normal limits for LVWT in both males and females [20]. In a comparison between highly trained Caucasian and African/Afro-Caribbean male athletes, black athletes (BA) have been found to exhibit a greater LVWT [61, 62]. This is supported by others who found BAs to have greater LVWT and LV mass compared to WAs (9.2 ± 1.2 mm vs. 8.6 ± 1.2 mm and 187.2 ± 42 g vs. 172.3 ± 42 g respectively) [63]. Furthermore, in male athletes it has been found that 12.4% and 18% of BAs presented a WT > 12 mm, compared with 1.6% and 4% of WAs whilst 3% of BAs presented a WT ≥ 15 mm compared with none of the WAs [62, 64]. Left ventricular WT values exceeding 15 mm in BAs should raise suspicion of pathology and therefore warrant further investigation [65]. BAs also exhibit a more pronounced training response compared to their white counterparts [62].

A higher prevalence of LVH and increased LVWT in adult male, female and adolescent BAs when compared with their white counterparts has also been demonstrated [63, 64, 66–71]. It should be noted that 3% of adult female BAs had a LVWT > 11 mm compared to none of the female WAs [63]. The prevalence of LVH however, was found to be lower in both white and black female athletes when compared to black and white male athletes [66]. In a recent study, it has been found that adult male BAs are more likely to exhibit concentric remodelling or hypertrophy, and have a significantly higher left ventricular mass index (LVMI) in comparison to their white counterparts (104.6 ± 24 g/m^2^ vs. 101 ± 21 g/m^2^ respectively) [72]. This study also provided important findings regarding cardiac remodelling in female BAs, highlighting similar geometry to male BAs but with the majority of athletes demonstrating normal LV geometry [72]. The study also concluded that the physiological cut off for relative wall thickness (RWT) in BAs is higher than would be considered for WAs with 0.48 in females and 0.51 in males [72]. However, the BAs and WAs in this study were from different sporting disciplines and hence this may, in part, explain the disparity observed.

Systolic blood pressure at peak exercise has been found to be a predictor of LV mass and a study found adolescent BAs exhibit a higher systolic blood pressure during exercise compared to WAs and, hypothesised this could be a mechanism underpinning a more pronounced LVH response [64, 67].

In a recent review, athletic training has been found to be associated with increased LV trabeculation and can therefore overlap with criteria for LVNC [20]. It has also been found that LV trabeculation and hypertrabeculation are more prevalent in African/Afro-Caribbean athletes when compared to their white counterparts, and specifically in athletes who take part in high-dynamic sports such as soccer and running (hypertrabeculation; 28.8% vs. 16.3% respectively) with more of these athletes meeting two criteria for LVNC diagnosis [64, 73]. This increased trabeculation however, although seemingly a physiological process of the black and white AH, appears to be more challenging in the black athletic population when aiming to differentiate between normal physiology and LVNC [20, 73].

##### West Asian athletes

Compared with sedentary controls, Male Arab athletes demonstrate increased LV mass and LVWT [64]. However, the differences in LVWT of the Arabic/Middle Eastern AH are similar when compared to differences in the WA (0.5% vs. 0.6%) [64, 74]. This may be attributed to differences in body size between WAs and the smaller Arabic/Middle Eastern athletes as these differences are negated when scaling to BSA [20, 74]. Additionally, global measures of LV function seem to be similar across all groups with all ethnicities presenting normal systolic and diastolic function [20].

##### South and far east Asian athletes

Compared to other ethnicities, research governing LV adaptation in Asian athletes is scarce. However, both male and female Chinese athletes have been found to display a similar prevalence and magnitude of LV cavity dilatation and hypertrophy compared with their white counterparts [75]. However, eccentric LV remodelling has been found to be more common in Japanese athletes compared to BAs and WAs, demonstrating an enlarged LV cavity when scaled to BSA [76]. Furthermore, on the more extreme end of the spectrum, structural morphology has been assessed in a group of Japanese ultramarathon runners [77]. Extreme LV dilatation (LVIDd ≥ 70 mm) in 11.3% of their study population was reported, with a LVWT of up to 19 mm [77], although there is a lack of comparable data. Additionally, we cannot confirm whether these findings are due to a consequence of ultra-endurance training and whether they pose any clinical significance for diagnosing pathology.

##### Other ethnicities

Data on other ethnic groups are scarce. Pacific Islanders are indigenous people from Melanesia, Micronesia, and Polynesia. In a study examining Pacific Islander rugby league players, they were found to have an increased LV mass and RWT when compared to white players [78].

Adolescent mixed ethnicity footballers, specifically African and Caucasian descent, present phenotypical similarities with BAs with significant increases in LVWT compared to WAs but a smaller magnitude when compared to BAs [64, 79].

#### The right ventricle

Studies have demonstrated that RV structural adaptation in BAs and mixed ethnicity are similar to WAs [80, 81], with the upper limits for RV size being applicable across ethnicities and irrespective of body size. That aside, others have reported that the impact of ethnicity has minimal impact on RV adaptation and hence race-specific RV reference values can be deemed unnecessary [81, 82]. Further research is required to definitively establish the impact of ethnicity on RV structure and function.

It is of interest that if AVC criteria are considered, 9.9% of adolescent black footballers fulfil the structural criteria for definite or borderline AVC diagnosis whereas this is less likely in mixed ethnicity and WAs (3.9% and 0.6% respectively) [80]. Right heart size in excess of standard adult ranges were found to occur in as many as 1 in 22 adolescent athletes and therefore it would not be unusual to observe values that overlap with AVC structural diagnostic criteria [80].

#### The atria

To the best of our knowledge there is only one study that has assessed LA dimensions in African/Afro-Caribbean athletes. Compared to WAs, BAs have larger LA dimensions although the clinical significance of this finding remains unclear [71]. Furthermore, there are no data pertaining to other ethnic groups and, therefore, our existing normal ranges should be applied to all ethnicities until further research has proven otherwise.

#### The aorta

Whilst there are no studies conducted on the aorta between athletes of different ethnicities, there is evidence to suggest that the size of the aorta differs between ethnicities in non-athlete populations with black ethnicities having smaller BSA-indexed ascending aortic dimensions compared to white ethnicities [83] (Table 2).

**Table 2:** Key consideration points regarding ethnicity for preparticipation screening

Key points for preparticipation screening
There is a greater prevalence of concentric LVH in BAs compared to WAs, Asians, Arabic, Pacific Islanders, and mixed ethnicity
MEAs have phenotypical similarities to BAs although less pronounced and have a greater LVWT than WAs
BAs who have a RWT of up to 0.51 in males and 0.48 in females may be physiological although this should be interpreted with caution in the presence of symptoms or a family history of SCD
RV structural adaptation is similar in BAs, WAs and MEAs
BAs appear to have larger LA dimensions than WAs but this needs to be reproduced in future studies and until then the existing normal ranges should be applied

### Body size

#### Left ventricle

It is well established that heart size is dependent on body size, and it is convention in echocardiography to scale structural indices to a relevant body size scalar [84]. Due to the ease of access to height and body mass, derived BSA is the most common scalar for this purpose and traditionally in both athlete and non-athlete populations, cardiac chamber size is scaled ratiometrically to BSA based on the assumption that the relationship between body size and cardiac size is linear. The laws of geometric similarity dictate that most biological relationships are allometric and therefore indexing should occur by dividing the chamber of interest by the scalar raised to a population derived allometric exponent (*b*), hence refuting the first assumption. Many AH studies have sought to formulate a suitable *b* exponent to facilitate the comparison of LV mass between different studies and to accurately remove the impact of body size [20, 84]. Using the example of height, the *b* exponent values generated differ between 1.97 and 3, with variances in cohort sex, age and physical fitness [84]. However, the challenge of producing a *one-size-fits-all* value has been highlighted via a similar range of *b* exponents being described for indexing to body mass [20].

In addition, it has been proposed that BSA is an appropriate scalar to represent muscle mass i.e., metabolically active tissue. Generally, in an athletic population, fat mass is low and hence body mass and derived BSA may act as a reasonable surrogate for fat free mass (FFM). However, in obese populations, fat mass significantly confounds BSA. Studies have therefore produced data pertaining the efficacy of FFM as an indexing variable [14, 53, 85–88]. It has been found that LVEDD and LV mass are predicted by FFM and that ratiometric scaling of these variables to FFM provided a stronger correlation than BSA or height^2.7^ [88]. Furthermore, when indexed to FFM, there were no training-related differences observed; suggesting the extent of LV remodelling of the AH may reflect a normal physiological response to the increased FFM induced by training [85, 88]. These findings have been reproduced in more recent studies. In both football and rugby players, FFM has been identified as the most prominent indicator of LV mass [85, 87]. As the physiological adaptation of the LV appears to be proportional to body size and remain within normal limits, even in athletes displaying extreme anthropometry, scaling of LV structures to FFM appears to be optimal [20, 82]. This method therefore seems to overcome many of the limitations of extreme body anthropometry observed in athletes as LV mass and FFM develop synchronously [53]. Interpretation of those athletes who exceed the normal physiological limits during an echocardiographic assessment may benefit from the assessment of body composition for indexing to FFM [87].

In a study assessing asymptomatic athletes without a family history of SCD it has been found that BSA has a strong linear relationship with LV dimensions [82]. They discern that BAs have larger cardiac dimensions than white and Asian ethnicities and that for athletes with the largest BSA’s, black African ethnicity was associated with larger cardiac dimensions [82]. Drawing conclusions using data derived from WAs has the unsettling potential of generating false-positive diagnoses of pathology in the black athlete [62].

#### The right ventricle

Literature examining the scaling of RV structural parameters are scarce, which could be representative of the challenging geometry of the RV [20, 84]. George et al. found no significant linear relationships between body mass, BSA, height and RVIDd measurements [84]. In agreement with this are the findings that simple ratio scaling for RV dimensions to BSA did not show size independence in endurance athletes [37, 89]. The authors found that scaling for BSA allometrically produced size independence and therefore concluded RV size is allometrically related to BSA [37, 89]. This suggests that measurements of right ventricular outflow tract and RV length should be allometrically scaled with population-specific allometric *b* exponents [20, 37]. From a clinical perspective, use of these indexing methods may provide a greater efficacy in the identification of AVC. This is strongly recommended within the athletic population as the AH often exceeds expected values, thus falling into the ‘grey area’ of AVC pathology and normal athlete physiology.

#### The atria

To the authors knowledge, only 2 studies have examined scaling of the athlete’s LA, with no data governing indexing of the RA. The LA appears to conform to conventional geometrical scaling similar to the LV [84]. Also observed was a significant linear relationship between LA linear dimension and height when utilising ratiometric scaling [84]. More recently, it has been found that indexing LA linear dimension to BSA with a population-specific *b* exponent provided more acceptable body size-independent values [90]. Furthermore, it has been discerned that in contrast to BSA, lean body mass leads to body size independent scaling which may be especially important in screening athletes who present with very low body fat [90].

#### The aorta

To the best of our knowledge there are three studies pertaining to the effects of body size on aortic dimensions in athletes. Indexed aortic root size have been shown to have correlations with ratiometric scaling by BSA and LV mass, with greater values in men compared with women [55, 91]. It should be noted however, that aortic root size falls within established normal limits for the general population and therefore for athletes who exceed these values, indexed aortic references ratiometrically scaled to height may be helpful in the early detection of aortic pathologies [55, 59, 91] (Table 3).

**Table 3:** Key consideration points regarding body size for preparticipation screening

Key points for preparticipation screening
Indexing LVEDD and LV mass to FFM is optimal compared to BSA, body mass and height and in athletes displaying extreme anthropometry. Indexing to BSA with population-specific allometric *b* exponents, however, is also valid, and yields a greater ease of access
RV size is allometrically related to BSA when indexed with population-specific allometric *b* exponents. This may improve the efficacy in AVC identification in athletes who present with extreme anthropometry
LA diameter and height appear to have a significant linear relationship when ratiometric scaling is utilised. Lean body mass may be especially important in screening athletes with low body fat
Indexed aortic root dimensions have correlations with ratiometric scaling to height. As aortic root diameter values typically fall within established normal limits, indexed aortic reference values may be helpful in the early detection of aortic pathologies in athletes who exceed these limits

### Sex

#### The left ventricle

Female athletes consistently display smaller LV cavity dimensions and WT compared with their male counterparts [90, 92, 93]. Additionally, LV hypertrophy in female athletes (WT > 11 mm) is extremely rare when compared with their male counterparts and appears to be significantly blunted i.e. reaching maximum hypertrophy after 3 months when engaging in the same training as males [94, 95]. Furthermore, within this male athlete cohort, between 2.5% and 5% present a WT exceeding 12 mm [95]. A more recent study also found that although female athletes engaging in skill, power, endurance and mixed Olympic disciplines, they had smaller absolute LV dimension values, when they indexed these findings to body size they were greater when compared to males [25]. The authors went on to find that females also had significantly higher LV/RV ratio (1.41 ± 0.16 vs. 1.36 ± 0.15) and a lower RV outflow/inflow ratio (1.31 ± 0.23 vs. 1.43 ± 0.23) when compared to their male counterparts [25]. Although this is a single study it suggests divergent cardiac remodelling in female athletes compared with males. In contrast, larger LV dimensions have been reported in competitive male cyclists despite indexing for BSA, BSA^0.5^ and FFM^−1^ [93]. These conflicting data may be attributable to the different scaling approaches used but does raise interest regarding potential sex related differences in response to exercise training.

Global systolic function is analogous between female and male athletes, with disparities in absolute LV stroke volume being removed when scaling to FFM [90]. Additionally, two studies reported higher LV GLS in female ET athletes when compared to ET males with the former also reporting a slightly higher LV EF (66% vs. 63%) [90, 96]. This is consistent with what is known in the non-athletic population, however, it does not translate into clinically significant differences in systolic longitudinal strain rate or LV stroke volume index [90, 96].

Studies have also attempted to highlight differences in diastolic function but these are unremarkable with exception of a single study that highlighted a lower early diastolic longitudinal strain rate in males compared with their female counterparts (1.56%/s vs. 1.815/s) [90]. Further research is required in a significantly larger cohort to discern whether these findings are reproducible.

#### The right ventricle

Much like the LV, smaller RV structural dimensions are observed in female athletes when compared with males [25, 90]. These intersex differences in chamber dimensions can be removed by indexing to FFM suggesting that these differences are due to disparities in body size between sexes [90]. Also observed was a lower early diastolic longitudinal strain rate in the RV free wall in ET and RT male athletes when compared to sport-matched female athletes, suggestive of a slightly enhanced diastolic function at rest in the female athlete [90].

Long-term ET promotes an increase in all RV indexed dimensions and induces a more spherical RV shape in both male and female athletes [96]. This study also found that there were no significant differences in RV volumetric function between male and female ET athletes with tricuspid annulus velocity ratios being also similar between sexes [96]. Both males and female athletes have also been shown to have lower RV basal segmental strain values when compared to controls [96], with male athletes having larger RV cavities and lower bi-ventricular global strain compared to females [90, 96].

#### The atria

Training-induced biatrial dilatation has been observed in male and female athletes, with larger absolute dimensions being displayed in male athletes [20]. In addition, the relative magnitude of physiological adaptation in LA dimensions was similar between sexes [20] albeit with female athletes having greater bi-atrial reservoir strain [97]. Conversely similar LA volumes and reservoir strain were observed between ET males and females at rest whilst female athletes had lower RA volumes [98]. Interestingly, in highly trained ET athletes, females have similar or sometimes even a lower magnitude of atrial remodelling compared to males but with a better functional capacity based on reservoir and conduit strain [98]. These heterogeneous findings require further refined studies to elicit the impact of sex on atrial adaptation.

#### The aorta

In highly trained competitive athletes, males have been found to have larger aortic root dimensions than females [91, 99, 100]. Additionally, it has also been found that across 28 endurance, power and skill disciplines aortic root sizes of ≥ 40 mm for males and ≥ 34 mm for females were present in 1.3% and 0.9% respectively [100]. These findings provide useful insight that these abnormal values (≥ 40 mm for males and ≥ 34 mm for females) are unlikely caused by physiological responses to training [100, 101] (Table 4).

**Table 4:** Key consideration points regarding sex for preparticipation screening

Key points for preparticipation screening
Concentric LVH is extremely rare in female athletes and rare in male athletes
Female athletes present with smaller LV, RV and bi-atrial structural dimensions
Males have larger aortic root dimensions compared to females with values of ≥ 40 mm for males and ≥ 34 mm for females being extremely rare. Values exceeding these limits may be indicative of pathology and further assessment would prove beneficial

### Age

#### The left ventricle

In adolescent athletes of variable ethnicity, LVWT, LVEDD, left ventricular end diastolic volume index and LV cavity enlargement are greater when compared to age-matched controls [68, 69, 102–108]. Adolescent male athletes have a greater LV mass and WT compared to their female counterparts with 35% of males and 25% of females presenting WT values outside of paediatric reference values [108–110]. The enlargement of the LV cavity very rarely exceeds 60mm in adolescent males but in cases where it does, whilst also in the presence of an impairment of systolic or diastolic function, a diagnosis of DCM should be considered [103]. Furthermore, only a small proportion of adolescent athletes partaking in ball, racket and endurance disciplines exhibit LVWT values (> 12 mm in males & > 11 mm in females) [107].

Exercise training has a more profound effect on the adolescent BAs heart compared to WAs or mixed ethnicity [79, 98]. 7% of BAs compared to 0.6% of WAs presented LVH on their echocardiogram and with the very young (< 16 years) 5.5% presented LVH compared to none of the WAs [108]. Interestingly, BAs as young as 14 years old can exhibit LVWT values of 15 mm [108]. These structural adaptations in the adolescent BA appear to be a specific phenotype already present in pre-adolescence suggesting ethnic and genetic factors play a pivotal role in LV remodelling in the early years of life [68].

Senior athletes demonstrate a more pronounced enlargement of the LV cavity and increases in WT due to a greater physical maturity and increased cumulative training hours compared to the adolescent athletes [20]. A lifelong exercise dose–response relationship with cavity size has been demonstrated [111]. Significantly higher LVEDV values were observed in masters athletes when compared to age-matched casual exercisers and sedentary controls [111]. Senior ET athletes have been found to have greater LV mass and volumes compared to age-matched sedentary controls with no signs of LV dysfunction [112]. It has also been found that more than 10 years after cessation of training, some structural adaptation remains [111].

There are no differences in EF between masters athletes, sedentary older individuals, junior athletes, sedentary young individuals and pre-adolescent athletes [103, 107, 113–116]. However, some young competitive athletes (< 35 years) may present with lower EF of < 52% [117]. Additionally, between pre-adolescent football players and age-matched controls, there were no differences in peak GLS or systolic strain rate (SSR) suggesting training status had no effect on intrinsic contractility in this population [115]. GLS and strain rates have been found to be greater in athletes compared to untrained controls suggesting an increased systolic function whereas a recent study found GLS to be mildly reduced in ET athletes (aged < 35 years) when compared to sedentary controls and may be explained by the heterogenous presentation of the athletic population [116, 118]. These findings may also be directly related to age as GLS has been found to decrease with increasing age in healthy individuals [119].

Global diastolic function is similar between age-matched controls and junior athletes [103, 107]. A recent review has demonstrated that long term exercise does not prevent the gradual slowing in the rate of relaxation in resting global diastolic function that has been associated with increases in age [113]. Nevertheless, masters athletes have been found to have superior diastolic function with greater E and e′ velocity and lower A and a′ velocity, resulting in greater E/A and e′/a′ in comparison to sedentary controls [113]. This superior diastolic function may represent beneficial intrinsic relaxation but could also be related to bradycardia in the athlete, thus lengthening the diastolic period [113].

#### The right ventricle

Similar to that of the LV, RV cavity size is observed to increase throughout adolescence in young athletes due to both increased training volume and maturation [84, 120, 121]. Furthermore, in a study comparing senior and academy footballers with sedentary controls, both academy and senior players had larger scaled RV structural parameters compared to sedentary controls with senior players having larger RV dimensions than academy players [122].

RV systolic strain rate in the mid and apical wall has been shown to be reduced in adolescent athletes when compared to older athletes and sedentary controls, suggestive of RV systolic reserve, whilst RV S’ and RV peak strain have been found to decrease with age [121–123]. Furthermore, in a study comparing academy footballers to their senior counterparts, systolic strain rates in the mid and apical walls were lower in the senior players compared to the academy players [122]. In ET children (10.8 ± 0.2 years), RV function remained normal, with no changes in RV GLS, RV S’ values and fractional area change [120].

To the authors’ knowledge, only two papers describe the effects of age on RV diastolic function. It has been found that with increasing age of the athlete, e` and the trans-tricuspid E/A ratio decline with the ratio being coupled with a decreased ability to augment diastolic function during exercise stress [117, 123].

#### The atria

LA cavity size, bi-atrial volumes and RA area have been found to be greater in adolescent athletes compared to sedentary controls [84, 102, 106]. Additionally, this training induced remodelling of the atria has been found to also be associated with a preserved biatrial function measured volumetrically, with LA and RA EF being similar between ET child athletes and age-matched sedentary controls [102]. Furthermore, lifelong endurance athletes engaged in marathon running have also been found to display significantly larger atria compared with sedentary controls [124]. To the best of our knowledge there are no data pertaining to atrial function in older athletes and therefore future research to help develop our understanding of this area will be valuable. Future research comparing atrial structure and function between adolescents, middle-aged and older athletes will also prove useful as it will help further our understanding of the effects of lifelong exercise on atrial adaptation.

#### The aorta

Aortic diameter is greater in adolescent athletes compared with age-matched sedentary controls [54, 125, 126]. In a recent study, aortic dilation was found to be present in 6.4% of the screened athletes (aged 14–34), but this may be related to a lack of age-specific cut-off values. [54, 125, 126] (Table 5).

**Table 5:** Key consideration points regarding age for preparticipation screening

Key points for preparticipation screening
LV dimensions in adolescent athletes are larger when compared with sedentary controls. LV cavity enlargement rarely exceeds 60mm but in cases where it does, whilst also in the presence of an impairment of systolic or diastolic function, a diagnosis of DCM should be considered
Senior athletes present more pronounced LV and RV dimensions compared to adolescent athletes and sedentary controls, due to their increased physical maturity and greater cumulative training hours
Adolescent athletes present bi-atrial remodelling compared to sedentary controls. However bi-atrial function is preserved with LA and RA EF similar between athletes and controls and thus signifies normal physiological remodelling
Aortic dilation is rare in adolescent athletes. The aortic diameter cut off values of 40 mm for males and 34 mm for females may not be appropriate for the adolescent athlete and therefore scaling to height is warranted

### Deconditioning and technical considerations

It has been reported that in elite and Olympic athletes who withdraw from training a reduction in RV and LV cavity dimensions, mass and WT occur, which can therefore be suggestive of a reversibility of any hypertrophy that is induced by athletic training [127–129]. Furthermore, a recent narrative review discerns that although cardiac atrophy is observed, even with short periods of detraining (1–8 weeks), systolic and diastolic function of the heart appear to be spared [129]. However, there are important implications for distinguishing between normal AH physiology and pathology. It is suggested that if cardiac atrophy and reductions in WT are present after cessation of training, then the hypertrophy that was present was likely an adaptation to athletic training and not that of pathology [127]. This could prove particularly useful in ambiguous cases in which the clinician is uncertain whether the athlete has training induced hypertrophy, and thus normal physiology, or whether the athlete is presenting with cardiomyopathy.

The assessment of LV mass is fundamental to defining geometry in the AH and yet the validity and reproducibility of echocardiographic is debatable with the use of different methods and modalities i.e. m-mode versus 2D. It is therefore important to acknowledge the variability applied to the empirical studies seen presented in this literature review (see Table 6). It is likely that future studies should aim to standardise methodology as well as consider other modalities, such as 3D echocardiography, where LV mass is more closely related to that measured by cMRI [130]. In this regard it is vital that other indices of cardiac structure and function are carefully made and follow specific BSE protocols in order to reduce intra and interobserver variability [131, 132].

**Table 6:** Methods used in each study for assessing LV mass

Empirical study	Method used
Bjerring et al. (2020)	Not stated
D’Andrea et al. (2010)	Not stated
D’Andrea et al. (2013)	Penn convention
Kleinnibbelink et al. (2022)	Disk summations technique
Pelliccia et al. (1999)	Not stated
Spence et al. (2011)	Not stated
Baggish et al. (2008)	Area-length
Au et al. (2019)	Not stated
Forsythe et al. (2018)	ASE equation
Dores et al. (2018)	Not stated
Huang et al. (2019)	Not stated
Rawlins et al. (2010)	Devereux
Basu et al. (2018)	Not stated
Demola et al. (2019)	Devereux
Galanti et al. (2019)	Devereux
Malhotra et al. (2018)	Not stated
McClean et al. (2023)	Not stated
Basu et al. (2023)	Not stated
Ma et al. (2007)	Devereux
Johnson et al. (2017)	ASE equation
Malhotra et al. (2017)	Not stated
George et al. (2001)	Devereux
Rato and Richards (2022)	Not stated
Martinho et al. (2020)	Devereux
Whalley et al. (2004)	ASE equation
Giraldeau et al. (2015)	Area-length
Pelliccia et al. (2023)	Not stated
Rowland and Roti (2010)	Devereux
D’Ascenzi et al. (2016)	ASE equation
Makan et al. (2005)	Devereux
Rodriguez-Lopez et al. (2016)	Not stated
Rundqvist et al. (2016)	Linear method at the parasternal long axis approach
Sharma et al. (2002)	Devereux
Sheikh et al. (2013)	Devereux
Forså et al. (2023)	Devereux
Pelà et al. (2016)	Penn convention
Missenard et al. (2019)	Not stated
Maron et al. (1993)	Devereux
Pelliccia et al. (2002)	Devereux

### COVID-19 infection and cardiac involvement

Myocarditis plays a crucial role in the pathogenesis of SCD within the athletic population [133]. Dangerous arrhythmias can be triggered by physical exertion which can potentially advance myocardial damage in athletes with myocarditis [133]. Furthermore, myocardial inflammation caused by myocarditis can lead to ventricular arrhythmias and can also be associated with early LV systolic dysfunction [133, 134]. COVID-19 may predispose the athlete to myocarditis, and hence screening protocols assessing athletes recovering from COVID-19 is important [133].

Most studies utilise a combination of ECG, echocardiography, and cardiac magnetic resonance (CMR) to assess athletes who have tested positive for COVID-19. Echocardiography is a vital diagnostic tool in initial return to play assessment post-infection. This is due to its efficacy in evaluating global LV function, localized wall motion abnormalities, valvar dysfunction, and pericardial effusions [135]. If any cardiac abnormalities are identified on the echocardiogram, further imaging is indicated highlighting it as an important early diagnostic tool [135].

There is a consensus that athletes appear to have an overall low risk of Covid-19 induced cardiac abnormalities. These cardiac abnormalities—myocarditis, pericarditis and pericardial effusion—have been found to affect between 0.2% and 4% of athletes with a single study having a prevalence of 40% of athletes [136–141]. That aside, there are other data reporting an absence of COVID-19 induced cardiac involvement [142, 143]. However, these findings are most likely due to the wide heterogeneity in study designs and gaps in study population sizes. Although the risk of cardiac involvement appears low, post Covid-19 infection screening, particularly in those with moderate to severe symptoms clinically, may still prove beneficial in detecting any cardiac involvement.

In a study conducted on 455 football players, 3% demonstrated de-novo ECG changes post COVID-19 infection, with 88% of these athletes being diagnosed with cardiac inflammation [144]. Furthermore, these de-novo ECG changes were able to detect an additional 20% of athletes with cardiac inflammation who presented with non-cardiac symptoms [144]. Taken with previous studies there is an agreement for a low risk of cardiac pathology post COVID-19 infection but there are still athletes presenting with inflammation. This is particularly worrisome due to the complications these abnormalities induce on the cardiac chambers including LV arrhythmias and a reduced early LV systolic function [145]. Further research is required to determine the function of the AH post Covid-19 infection in large heterogenous populations. The effects of emerging variants of the virus and long-term outcomes are still unknown and will provide useful knowledge for the practitioner as more research is forthcoming. Until then, appropriate cardiac screening is imperative for athletes with a higher pretest probability for myocarditis as well as any athlete that develops the virus [145]. For those athletes diagnosed with any cardiac abnormality, further screening will be vital in avoiding fatalities and contributing to a shared decision on the safe amount of physical activity (Tables 7 and 8).

**Table 7:** Key consideration points regarding COVID-19 infection for preparticipation screening

Key points for preparticipation screening
Echocardiography should be utilised as one of the first diagnostic tools of assessment after Covid-19 infection. The efficacy of this screening method in identifying cardiac abnormalities will aid the clinician in identifying whether further investigations are required
Covid-19 infection appears to have a low risk of inducing cardiac inflammation in athletes. In those athletes who have functional abnormalities consistent with inflammation via echocardiography, further screening and investigations are vital in avoiding SCD

**Table 8:** Summarisation of findings for each cardiac chamber

	Sporting discipline and training volume	Ethnicity	Body size	Sex	Age
Left ventricle	Endurance training elicits eccentric hypertrophy Concentric remodelling is relatively rare in any athlete Athletes with high levels of training volume show a more pronounced structural remodelling HIIT induces greater increases in mass Endurance athletes present with lower resting ejection fraction	There is a greater prevalence of hypertrophy in black athletes compared to athletes of any other ethnicity Trabeculation and hypertrabeculation are more common in black athletes	Indexing LVEDD and mass to FFM is most optimal compared to BSA, body mass and height and particularly in athletes displaying extreme anthropometry	Concentric hypertrophy is extremely rare in female athletes Female athletes have smaller structural dimensions than male athletes Female athletes present with slightly higher ejection fraction	Senior athletes > 60 years demonstrate a more pronounced enlargement of the cavity and increases in WT compared to adolescent athletes Adolescent black athletes have a higher presentation of LVH compared to age-matched white athletes
Right ventricle	Endurance trained athletes present balanced increases in mass and volume Resistance trained athletes present similar chamber dimensions to sedentary individuals Global resting function is maintained	RV structural adaptation is similar between ethnicities	RV size is allometrically related to BSA when indexed with population-specific allometric b exponents	Female athletes have smaller structural dimensions When indexing to FFM these intersex chamber dimension differences are removed suggesting body size is the cause of the disparities in size	The cavity size increases throughout adolescence and throughout physical maturity Systolic strain rate in the mid and apical wall is reduced in adolescent athletes suggestive of systolic reserve
Atria	Endurance training elicits bi-atrial dilatation and increased atrial volumes which are strongly correlated with exercise capacity	Black athletes appear to have larger LA dimensions than white athletes	LA diameter and height appear to have a significant linear relationship when ratiometric scaling is utilised Lean body mass may be especially important in screening athletes with low body fat	Males have larger absolute bi-atrial dimensions	Adolescent athletes present bi-atrial remodelling compared to sedentary controls Bi-atrial function is preserved with LA and RA EF being similar between athletes and controls
Aorta	Increased aortic root dimensions may be present in some athletes, but values usually fall within normal cut offs		Indexed aortic root dimensions have correlations with ratiometric scaling to height	Male athletes have larger aortic root dimensions	Aortic dilation is rare in athletes irrespective of age

## Conclusions

The AH is a complex phenotype with unique structural and functional characteristics as determined by sporting discipline, training volume, ethnicity, body size, sex, and age. Identifying normal physiology for each individual athlete is imperative when undertaking preparticipation echocardiographic screening. Our understanding of normality helps to improve sensitivity and specificity for the detection of cardiac disease and hence reduces anxiety or the unnecessary disqualification of an athlete, whilst ensuring that pathology is accurately detected. Furthermore, with the new complex challenge of COVID-19, understanding the AH is even more vital in protecting the health of our athletes.

## Data Availability

Not applicable.

## Abbreviations

AH: Athlete’s heart
AVC: Arrhythmogenic (Right Ventricular) cardiomyopathy
BA: Black athlete
BSA: Body surface area
CMR: Cardiovascular magnetic resonance
DCM: Dilated cardiomyopathy
e′: Early diastolic annular velocity
EF: Ejection fraction
ET: Endurance trained
FFM: Free fat mass
GLS: Global longitudinal strain
HCM: Hypertrophic cardiomyopathy
LA: Left atrium
LV: Left ventricle
LVEDD: Left ventricular end diastolic diameter
LVEDV: Left ventricular end diastolic volume
LVMI: Left ventricular mass index
LVNC: Left ventricular non-compaction
RFL: Rugby football league
RT: Resistance trained
RVD1: Rightventricular basal diameter
RVIDd: Right ventricular internal dimension in diastole
RVOT: Right ventricular outflow tract
RWT: Relative wall thickness
S’: Systolic annular velocity
SCD: Sudden cardiac death
TAPSE: Tricuspid annular plane systolic excursion
WA: White athlete
WT: Wall thickness
